# Quantitative Analysis of EEG Power Spectrum and EMG Median Power Frequency Changes after Continuous Passive Motion Mirror Therapy System

**DOI:** 10.3390/s20082354

**Published:** 2020-04-21

**Authors:** Taewoong Park, Mina Lee, Taejong Jeong, Yong-Il Shin, Sung-Min Park

**Affiliations:** 1 Department of Creative IT Engineering, Pohang University of Science and Technology (POSTECH), Pohang 37673, Korea; taewoongpark@postech.ac.kr (T.P.); minah@postech.ac.kr (M.L.); vical86@naver.com (T.J.); 2 Department of Rehabilitation Medicine, Pusan National University School of Medicine, Pusan National University Yangsan Hospital, Yangsan 50612, Korea; rmshin@pusan.ac.kr

**Keywords:** continuous passive motion (CPM), sensory stimulation, combination rehabilitation, mu rhythm, muscle fatigue, electroencephalogram (EEG) signal processing, electromyogram (EMG) signal processing

## Abstract

Robotic mirror therapy (MT), which allows movement of the affected limb, is proposed as a more effective method than conventional MT (CMT). To improve the rehabilitation effectiveness of post-stroke patients, we developed a sensory stimulation-based continuous passive motion (CPM)-MT system with two different operating protocols, that is, asynchronous and synchronous modes. To evaluate their effectiveness, we measured brain activation through relative and absolute power spectral density (PSD) changes of electroencephalogram (EEG) mu rhythm in three cases with CMT and CPM-MT with asynchronous and synchronous modes. We also monitored changes in muscle fatigue, which is one of the negative effects of the CPM device, based on median power frequency (MPF) and root mean square (RMS). Relative PSD was most suppressed when subjects used the CPM-MT system under synchronous control: 22.11%, 15.31%, and 16.48% on Cz, C3, and C4, respectively. The absolute average changes in MPF and RMS were 1.59% and 9.78%, respectively, with CPM-MT. Synchronous mode CPM-MT is the most effective method for brain activation, and muscle fatigue caused by the CPM-MT system was negligible. This study suggests the more effective combination rehabilitation system for MT by utilizing CPM and magnetic-based MT task to add action execution and sensory stimulation compared with CMT.

## 1. Introduction

Fifteen million people worldwide suffer a stroke each year. Even where advanced technology and facilities are available, 67% of those who suffer a stroke die or become permanently disabled. Approximately 50% of all stroke survivors remain physically disabled [1]. These consequences lead to a burden on families and communities. The majority of post-stroke patients are typically frustrated with both the immediate limb dysfunction and the ambiguity of how much motor control they can regain, which significantly impairs their quality of life as well as their willingness to rehabilitate. To revitalize self-supporting efforts for rehabilitation and improve quality of life, cognitive intervention plays an important role by helping patients have a more optimistic attitude toward their condition [2].

Mirror therapy (MT) is a cognitive intervention rehabilitation method for the recovery of the upper extremity function that produces a visual illusion in the brain that the paralyzed limb is fully functional by providing an image of the movement of the nonparalyzed limb through a mirror [3]. This method uses voluntary exercise to improve a patient’s motivation to rehabilitate [4] and prevents the deterioration of the motor ability of the lesioned brain hemisphere using a visual illusion that bilateral movement has been accomplished [5,6]. However, a major drawback of conventional MT (CMT) is that the paralyzed arm does not move at all during the treatment. Thus, muscle atrophy can be aggravated and the elbow can become stiff. Spasticity and hypertonus are likely to limit the functional use of the paralyzed limb. Additionally, muscle hyperexcitability may lead to secondary complications including contracture, pain, and reduced range of motion. In the long term, these complications may also result in structural changes in connective tissues and muscle fibers [7,8]. In such situations, patients lose their motivation to rehabilitate. In addition, the prevalence of strokes increases in an aging society, and the public healthcare burden of patients who have suffered a stroke increases. The current emphasis on cost reduction in medical services has resulted in reducing the duration of in-patient rehabilitation of post-stroke patients. This results in patients terminating treatment with incomplete recovery. These situations have triggered research on better and cost-efficient methods of treatment for post-stroke patients.

To overcome the current limitations of CMT methods, a robotic system that actually moves the affected limb has recently been proposed as an emerging rehabilitative method [9,10]. However, building a new system for only one rehabilitation method, like MT, is difficult to introduce in many hospitals owing to budget and utilization issues. In addition, introducing a completely new medical equipment to the real world clinical setting typically faces the immediate resistance for adoption. Therefore, we suggest using a continuous passive motion (CPM) device, which is already equipped in the hospital, can be used for other treatments, and is familiar to physical therapists, in order to increase the efficiency of MT. Then, this study was conducted as a pilot study to develop a closed-loop rehabilitation system for personalized treatment by analyzing the activation level of electroencephalogram (EEG) according to the rehabilitation protocol.

To the best of our knowledge, no study has aimed to improve MT efficiency in terms of brain activation using a CPM device. Therefore, we developed a customized CPM system to incorporate CPM into MT. Then, two different operating protocols of CPM-MT were designed: (1) the asynchronous mode, in which the paralyzed and nonparalyzed arms are controlled independently of time; and (2) the synchronous mode, in which the two arms are controlled simultaneously. To promote motor recovery, contemporary strategies focus on combination rehabilitation [11], which merges several treatments: voluntary movement, biosignal-based closed-loop systems, and sensory stimulation. In this flow, sensory input also plays an important role in post-stroke motor recovery [12,13]. Therefore, we added an MT task that can provide sensory stimulation. Additionally, changes in muscle conditions during rehabilitation must be monitored because the CPM device produces passive movements that can cause muscle fatigue [14].

## 2. Materials and Methods

### 2.1. Subjects

Changes in the brain that occur in post-stroke patients are similar to those that occur in healthy people when they actually/imaginably move their arms [15,16]. Therefore, we conducted experiments on healthy subjects.

Seven right-handed, healthy paid volunteers (five males, two females; mean age: 26.86 ± 3.39 years, range: 21–32 years) participated in this study. We recruited them from the Pohang University of Science and Technology (POSTECH), South Korea, via open recruiting. Subjects were excluded if they had any neurological disease, mental problems, history of brain tumor, stroke, head trauma, or musculoskeletal disease. The included subjects were instructed to abstain from caffeine and alcohol before recording, and they participated only once in each experiment. All subjects had a normal or corrected-to-normal vision and were told to assume that their left arm was paralyzed. Before the start of the experiment, all subjects gave written informed consent for participation, and the study protocol was approved by the Institutional Review Board of POSTECH.

### 2.2. Apparatus and Materials

**Figure 1:**
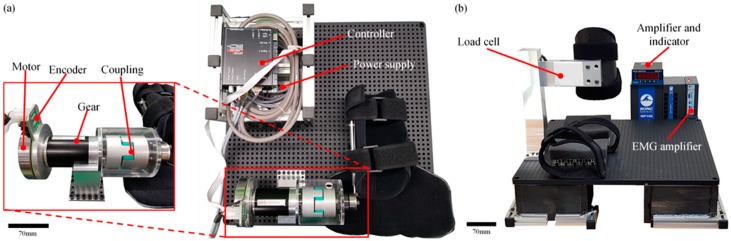
(**a**) The continuous passive motion mirror therapy (CPM−MT) system comprises coupling, gear, motor, encoder, controller, and power supply. (**b**) The muscle fatigue measurement system comprises load cell, load cell amplifier, indicator, and electromyogram (EMG) amplifier.

Experiments were performed with the custom-built CPM system for the rehabilitation of elbow flexion and extension shown in Figure 1a. The system comprised (1) a DC motor (EC90, Maxon motor, Switzerland), (2) a gear (GP52C, Maxon motor, Switzerland), (3) a controller (EPOS2 50/5, Maxon motor, Switzerland), (4) a power supply (NES-350-24, MEAN WELL, Taiwan), (5) a jaw type coupling for reducing mechanical shock, (6) a bearing for reducing rolling friction, (7) several structures of arm and motor supporters as well as the shaft, (8) a mirror, (9) a metronome (SQ200, SEIKO, Japan) for consistent timing, and (10) a personal computer that controlled the motor using LabVIEW 2013 (National Instruments, USA).

EEG signals were measured using an amplifier (WEEG-32, Laxtha, Daejeon, Korea), EEG electrodes (LXEL-SAF-DK, gold plated; Laxtha, Korea), and data acquisition software (TeleScan 3.03, Laxtha, Daejeon, Korea). A magnetic membrane comprising magnetic filler and silicone and a silicone gripper with a ball magnet were used for the elbow flexion and extension MT task, which attached the gripper to the magnetic membrane and took it off by elbow flexion. The magnetic membrane was fabricated by mixing silicone base 1 (Ecoflex 00-30, Smooth-On, Macungie, PA, USA), silicone base 2 (SH7340, XINUSLAB, Yangpyeong, Korea), a catalyst (SH7340 catalyst, XINUSLAB, Korea), and NdFeB powder (MQP-S-11-9, Neo Magnequench, Tianjin, China) as magnetic filler. The mixed material was poured into a mold, cured at room temperature, and cut into a suitable size for the experimental system. The gripper was made with a silicone base (Ecoflex 00-30, Smooth-On, Macungie, PA, USA), catalyst (Ecoflex 00-30 catalyst, Smooth-On, Macungie, PA, USA), and ball magnet (∅15 mm, JSMAGNET, Incheon, Korea). The gripper was ergonomically designed to be easily grasped by subjects and was made by placing the ball magnet at the center of the mold and then pouring the well-mixed material.

Muscle fatigue was measured (Figure 1b) with data acquisition hardware (MP160, BIOPAC, Goleta, CA, USA), an amplifier (EMG100C, BIOPAC, Goleta, CA, USA), three surface electromyogram (sEMG) electrodes (H124SG, COVIDIEN, Dublin, Ireland), data acquisition software (AcqKnowledge 5.0, BIOPAC, USA), load cell (CB5-300 kgf, DACELL, Cheongju, Korea), and indicator (DN50W, DACELL, Cheongju, Korea).

### 2.3. Customized CPM

**Figure 2:**
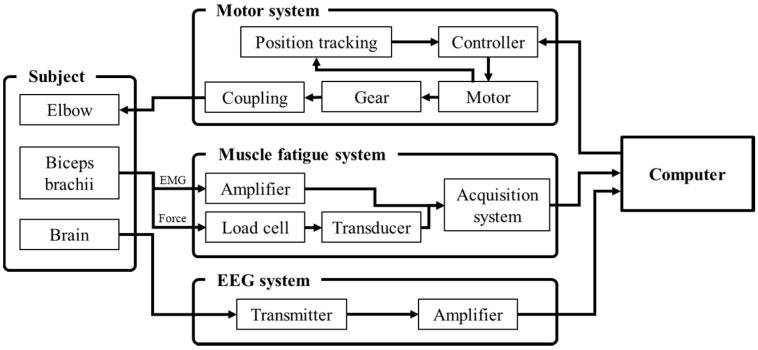
The continuous passive motion mirror therapy (CPM−MT) system overview.

The CPM-MT system generated the elbow motion of a subject’s left arm, and acquired biosignals from the biceps brachii and brain (Figure 2).

The torque of the CPM-MT system was generated by a geared direct current motor (EC90 with a gearhead of 113:1). The maximum available torque was 38.40 Nm, which was about three times larger than the maximum required torque based on American anthropometry statistics [17]. Jaw type coupling was used to alleviate the mechanical impact on the subjects of motor acceleration and deceleration.

The system repeated flexion and extension in the range of 0°–80° and was controlled independently of the intact arm at a velocity of 1000 rpm, an acceleration speed of 5000 rpm/s, a deceleration speed of 5000 rpm/s, and a time interval of 2 s (30 bpm).

### 2.4. Experimental Protocol

**Figure 3:**
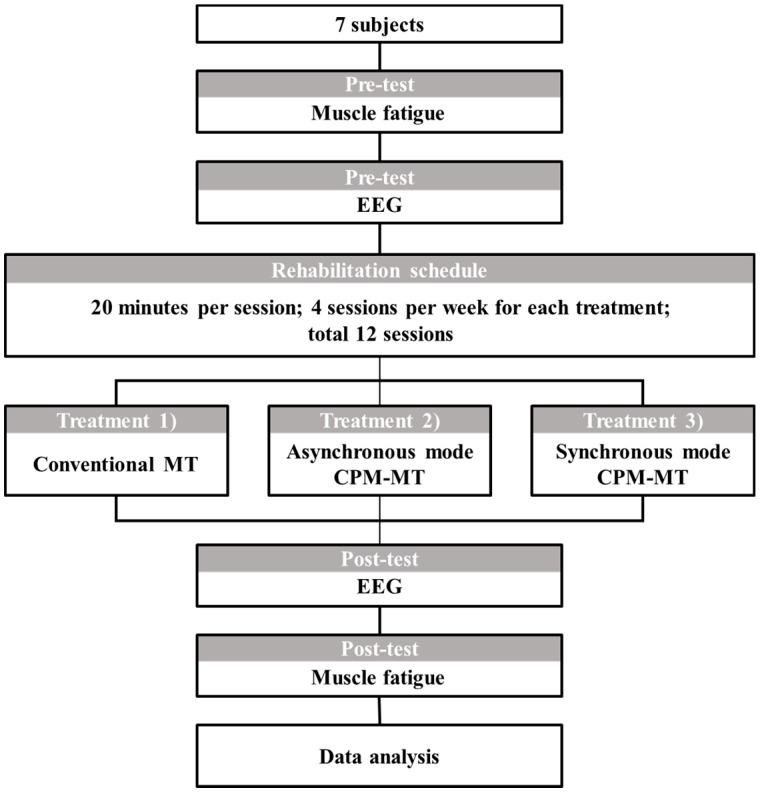
Experimental protocol.

The experiment proceeded as follows (Figure 3). Three sEMG electrodes (VIN+, VIN−, and GND) were attached to the left upper arm of the subject following the SENIAM guidelines [18].

**Figure 4:**
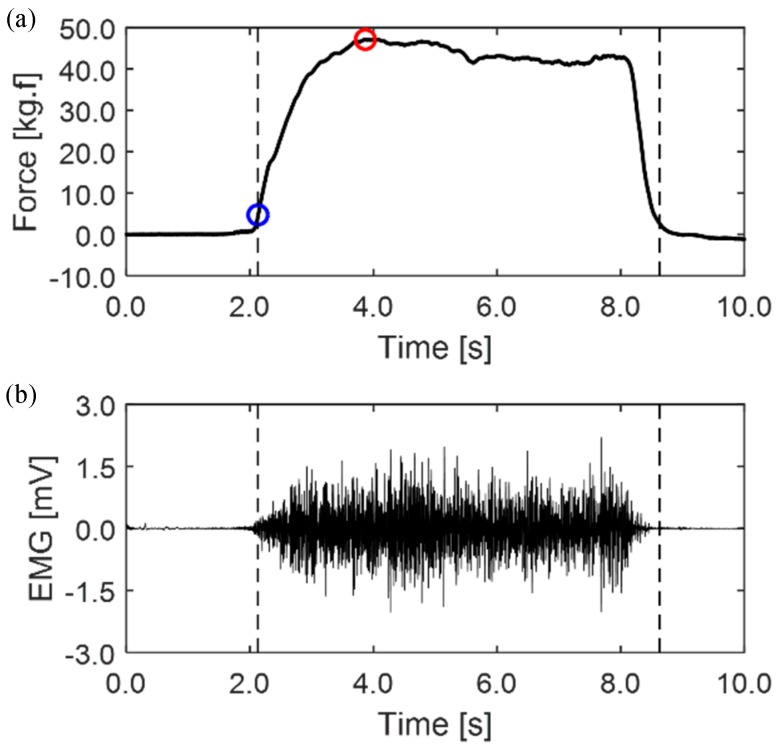
Example signal of muscle fatigue measurement. (**a**) load cell force data, red circle: 100% peak force, blue circle: 10% peak force. (**b**) biceps electromyogram (EMG) data.

Force data and EMG signal were measured for 5 s at 100% of the maximum voluntary contraction of the biceps brachii generated under voluntary isometric contraction (Figure 4); then, the subject rested for 3 min, and these procedures were performed five times [19,20,21].

Before the EEG electrodes were attached, a skin preparation gel (Nuprep, WEAVER, USA) was used to reduce skin impedance and increase conductivity between the scalp and electrodes; then, the electrodes were attached to the scalp using a conductive paste (Ten20, WEAVER, USA).

The disk EEG electrodes were placed according to the International 10-20 system of electrode placement on C3 (left central fissure), Cz (central fissure), and C4 (right central fissure). Reference electrodes were placed on the regions located behind each ear (mastoids). C3 and C4 are the primary sensorimotor areas, and Cz is a supplementary motor area [22,23]. These three areas are directly engaged in the process of planning and outputting exercise orders during motor activities [24,25].

EEG was measured with the subject in a resting state with their eyes open to monitor the changes from before to after the experiment. The subject sat in a relaxed position without any task for 6 min for the recording of pre-test EEG.

**Figure 5:**
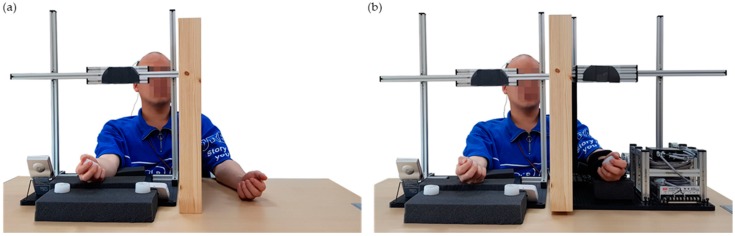
(**a**) Conventional mirror therapy (CMT) experimental setup. (**b**) The continuous passive motion mirror therapy (CPM−MT) system experimental setup.

Three types of operating protocol were conducted for 20 min each with the addition of a magnetic-based MT task as sensory stimulation to improve the effectiveness of rehabilitation: (1) CMT (Figure 5a), (2) asynchronous mode CPM-MT (ACMT), and (3) synchronous mode CPM-MT (SCMT) (Figure 5b). Next, post-test EEG was measured with the subject at rest without any task performed for 6 min. The previous force data and EMG signal measurements were repeated. This whole process was repeated four times for each treatment per week [26]. Thus, each experiment consisted of 12 measurements.

In protocol 1, that is, CMT without a CPM system, the paralyzed arm remained motionless and only the intact arm moved according to a tempo of 30 bpm of the metronome. Then, the subject observed the reflection of arm flexion and extension in the mirror.

In protocol 2, that is, ACMT, the system was controlled independently of the intact arm at a time interval of 2 s (30 bpm).

In protocol 3, that is, SCMT, the system was controlled by the timing at which the intact arm moved according to a tempo of 30 bpm of the metronome.

### 2.5. Signal Processing

All signal processing and analyses were performed using Matlab R2017b (MathWorks, Natick, MA, USA) and EEGLAB 14.1.2b toolbox [27].

#### 2.5.1. EEG Signal Processing

EEG signals were sampled at a frequency of 256 Hz, and 60 Hz power-line noise was removed using a hardware analog filter. The first and last minutes of the acquired signals were excluded. Rhythms recorded on C3, Cz, and C4 might be affected by the posterior alpha frequency band activity because the mu frequency band overlaps with the posterior alpha band, and the generator for the posterior alpha rhythm is stronger than that for the mu rhythm [28]. Then, the signals were high-pass filtered using a zero-phase Hamming windowed sinc finite impulse response filter with a cutoff frequency of 1 Hz using the eegfiltnew function of EEGLAB.

Next, artifact subspace reconstruction (ASR) was applied to remove high-amplitude artifacts (e.g., eye blink, muscle burst, and movement) [29,30]. Given an input of clean baseline data collected from the subject while standing still for 1 min, ASR identifies the regions of clean EEG within the data, from which it computes an unmixing matrix based on the geometric median. Principal component analysis was applied to the EEG data using sliding windows, thereby decomposing the data into subspaces, and the subspaces that deviate from the baseline are reconstructed with the unmixing matrix. We used nondefault parameters of a sliding window with a length of 250 ms and threshold of 5 standard deviations for the identification of corrupted subspaces without any channel rejection [31,32]. Next, inadequate EEG signals were rejected according to the following criteria: (1) signals with magnitude of <30 or >3000 µV and 2) signals with kurtosis of >5 standard deviations from the mean. Thus, there were no contaminated signals to analyze [33].

The value of mu rhythm suppression was calculated from the EEG signals measured while the subject was at rest with eyes open.

#### 2.5.2. EMG Signal Processing

EMG signal and force data were acquired using data acquisition hardware and sampled at a frequency of 2 kHz. Then, the EMG signal was filtered by a 60 Hz notch filter to remove line noise and a 5–500 Hz Butterworth fourth-order band-pass filter. The power spectrum [34] of the EMG data from signal onset, using threshold-based onset detection (10% of peak force), to 6.5 s, was selected by the force data of the load cell to calculate the median power frequency (MPF) [35,36]. Then, the root mean square (RMS) of the selected section of EMG signal was calculated.

### 2.6. Data Analysis

We tested the normality of the relative and absolute power spectral density (PSD) ratio. A paired t-test was performed on three protocols for each subject for relative PSD analysis, and the Wilcoxon signed-rank test was performed for absolute PSD analysis. MPF and RMS ratios were also tested to check normality; then, MPF and RMS data were analyzed by the Wilcoxon signed-rank test. All analyzes were performed with a 95% confidence level. Calculations were performed using SPSS 25.0.

## 3. Results and Discussion

### 3.1. Brain Activation Analysis: EEG

MT is a treatment based on neuroplasticity for recovering the lesioned brain. Therefore, we used EEG to verify the effect of MT in terms of brain activation [37].

The EEG mu rhythm is generated by the sensorimotor cortex and is prominently suppressed in subjects who execute and observe movements at frequencies between 8 and 13 Hz on Cz, C3, and C4 [38]. Thus, we measured and analyzed the data obtained at these three electrode positions [28].

To verify the changes in brain activation related to upper extremity function, we analyzed the relative and absolute PSD of EEG.

Relative PSD is defined as the PSD ratio of the specific frequency band of analysis target with that of the total frequency band. The benefit of the relative PSD is a reduction of the intersubject variance associated with absolute power, which arises from intersubject differences in the conductivity of the skull and scalp [39]. However, the disadvantage of the relative PSD from the definition is that alteration in only one frequency band of the denominator, total frequency band, affects the changes in relative PSD. For instance, although the power of the specific frequency band and analysis target remains unchanged, the increment in PSD of the other frequency band results in a decrease in relative PSD. Thus, it is difficult to accurately analyze the changes in the brain in terms of the specific frequency band based on relative PSD. Therefore, we analyzed both relative and absolute PSD for accurate analysis of the brain.

Additionally, we eliminated the first and last 1 min of each measurement to reduce the possibility of alpha modulation owing to the attention affecting mu power results [28].

#### 3.1.1. Relative PSD Analysis

The relative PSD changes of the EEG mu rhythm were used to reduce intersubject variance [39,40]. The change in relative PSD was calculated after four rehabilitation sessions using Equation (1):

(1) $$Relative\;PSD=\frac{PSD_{mu}}{PSD_{total}}$$

where the total frequency band of EEG comprises the theta rhythm (4–7 Hz), mu rhythm (8–13 Hz), beta rhythm (14–30 Hz), and gamma rhythm (31–50 Hz). The ratio of relative PSD was calculated using Equation (2):

(2) $$Ratio\;of\;relative\;PSD=\frac{relative\;PSD_{after}}{relative\;PSD_{before}}$$

#### 3.1.2. Absolute PSD Analysis

We performed an absolute PSD analysis to observe the changes in the mu frequency band. The change in absolute PSD was calculated after finishing the final rehabilitation sessions. The ratio of absolute PSD was calculated using Equation (3):

(3) $$Ratio\;of\;absolute\;PSD=\frac{absolute\;PSD_{after}}{absolute\;PSD_{before}}$$

#### 3.1.3. System Differences: CMT versus CPM-MT

**Figure 6:**
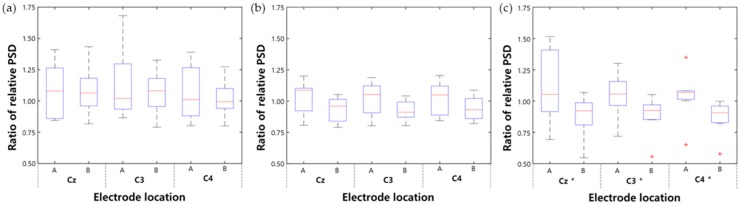
Ratio of relative power spectral density (PSD) for each type of treatment: (**a**) conventional mirror therapy (CMT), (**b**) asynchronous mode continuous passive motion (CPM)−MT (ACMT), and (**c**) synchronous mode CPM−MT (SCMT); (**A**) after first session and (**B**) after last session (* *p* < 0.05).

To compare the effects of CMT and CPM-MT, we first compared the CMT and ACMT groups (Figure 6a,b, Table 1). After a total of four rehabilitation sessions, the relative PSD of both groups did not show significant suppression of change after treatment (*p* > 0.05). Although we may have obtained these results via experiments with healthy subjects, it shows that developing the movement of the paralyzed arm without synchronizing the nonparalyzed arm does not affect brain activation for a short period of time.

**Figure 7:**
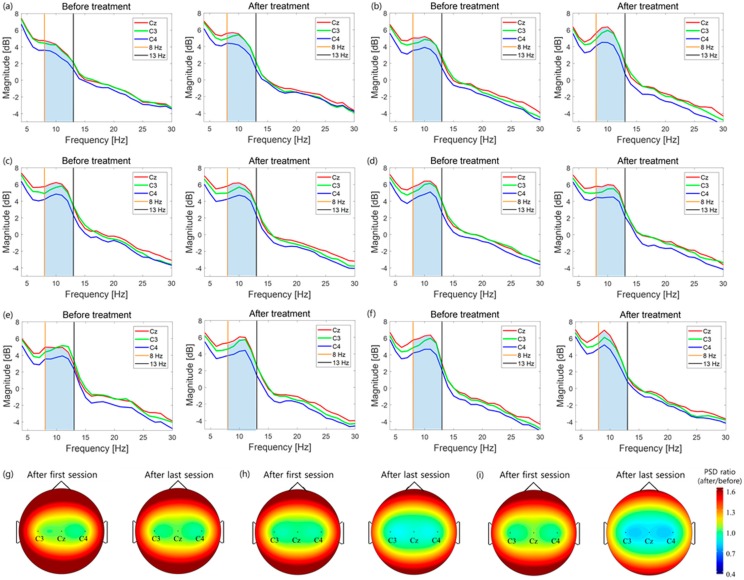
EEG analysis, (**a**) first session’s relative power spectral density (PSD), (**b**) last session’s PSD of conventional mirror therapy (CMT), (**c**) first session’s PSD, (**d**) last session’s PSD of asynchronous mode continuous passive motion (CPM)−MT (ACMT), (**e**) first session’s PSD, and (**f**) last session’s PSD of synchronous mode CPM−MT (SCMT); and the brain map of relative PSD ratio of (**g**) CMT, (**h**) ACMT, and (**i**) SCMT.

Regarding the change observed after each session (first and last), the relative PSD of both groups also did not show a significant difference (*p* > 0.05; Table 1, Figure 7a–d). This implies that there was no change in brain activation in the first and last experiments of CMT and ACMT.

**Figure 8:**
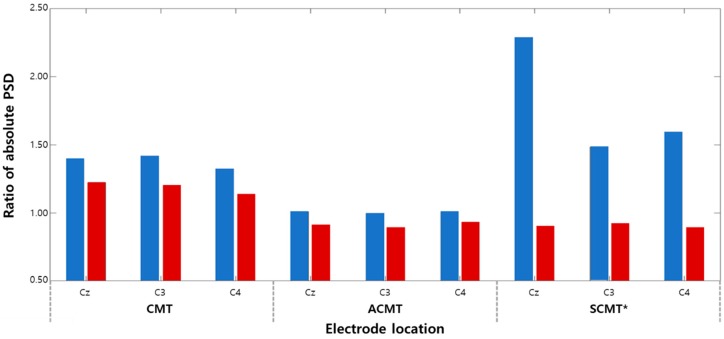
Absolute power spectral density (PSD) of the mu rhythm according to each rehabilitation protocol; blue: after first session, red: after last session (* *p* < 0.05).

In addition, changes in absolute PSD (Figure 8, Table 2) did not show significant difference in both groups after treatment (*p* > 0.05).

Comparison of the CMT and ACMT groups revealed that neither group suppressed the mu rhythm. One reason for this finding is that the healthy subjects possibly experienced an unnatural feeling during rehabilitation because the paralyzed arm did not move in CMT, and the movements of both arms were not synchronized in ACMT. Thus, we can conclude that CMT and ACMT cannot easily suppress the mu rhythm in a short period of time.

Next, comparison of the CMT and SCMT groups in terms of relative PSD showed that the change after treatment in SCMT showed significant suppression of the mu rhythm (*p* < 0.05; Table 1). This suggests that the mu rhythm, which is defined as an oscillation in the sensorimotor cortex, is suppressed more in SCMT than in CMT; thus, SCMT increases relevant brain activity.

In SCMT, the ratio of changes in relative PSD for each session (Table 1, Figure 7e–f) also showed significant suppression in the last session compared with that in the first session.

In addition, the absolute PSD of the mu rhythm significantly decreased in SCMT (*p* < 0.05; Figure 8, Table 2). This means that not only the relative PSD used to reduce the variance of the intersubject, but also the absolute PSD of the mu rhythm, is suppressed. Thus, these results indicate that the activity of the sensorimotor cortex increases in SCMT.

There is basic evidence suggesting that passive training may reorganize the cerebral cortex, providing evidence for the concept that proprioceptive training improves motor function [41]. Passive movements, with or without CPM system support, are a form of sensory stimulation (mainly proprioceptive input) that can activate the primary motor cortex (M1) and primary somatosensory cortex (S1) through two mechanisms: one is based on the overlap of M1 and S1 maps, whereas the other is based on the fact that M1 receives somatosensory input directly from the thalamus [42]. This suggests that proprioceptive inputs are part of the motor control network during the preparation and execution of movements [41,43,44]. Thus, SCMT may activate the brain by providing actual movement for stimulating the M1 region and the S1 region by providing the novel environmental (CPM assisted, synchronized control timing, and sensory stimulation) interactions in the short experimental period. In addition, SCMT may promote cortical plasticity by two mechanisms. One includes the simultaneous activation of both hemispheres, which is thought to facilitate the activation of the damaged hemisphere by reducing transcallosal inhibition from the unaffected hemisphere. In this respect, a rebalancing of interhemispheric inhibition would be enhanced [45,46,47,48]. The other mechanism involves palpation of the contralesional uncrossed corticospinal tract and spared indirect corticospinal pathways [6].

By these causes, SCMT stimulates M1 and S1 regions and promotes cortical plasticity, thereby suppressing the EEG mu rhythm. In contrast, CMT does not provide stimulation of the M1 region, and the S1 region may not be sufficiently stimulated by unnatural sensation. In addition, both hemispheres are stimulated disproportionately, so the neuroplasticity effect is not maximized. Next, in ACMT, the M1 region is stimulated, but the S1 region is not sufficiently stimulated owing to the unnatural sensation, and the balance between both hemispheres is not optimally matched because the movements of both arms are not synchronized, so the neuroplasticity effect cannot be maximized.

The results herein provide evidence that the SCMT can help restore motor function in post-stroke hemiplegic patients through brain activation. However, the present study has some limitations. Because the included subjects solely comprised healthy people, the possibility of generalizing the results to post-stroke patients remains unclear. Thus, further research is required. All subjects were consistently motivated to completely focus on experiments; however, it is difficult to control their concentration completely. Therefore, in future studies, it is necessary to monitor eye movements and judge complete participation in experiments. In a real clinical setting, CMT-based methods usually involve the motion of the hand or distal upper extremity, but our system implements two-dimensional elbow flexion and extension motion to control the experimental conditions. Therefore, the brain could be more activated if the system performed distal arm movement. EEG has a low spatial resolution; thus, differentiating the activity between the sensorimotor cortex and other regions is difficult. Thus, the mu rhythm may reflect both activity in the sensorimotor cortex and activity in other regions such as the superior temporal sulcus [49] and inferior parietal cortex [50,51], which are involved in action recognition. In future experiments, we can dissociate between these two sources of activation using high spatial resolution techniques such as functional magnetic resonance imaging (fMRI) and high-resolution EEG.

Despite these limitations, CMT, ACMT, and SCMT were conducted under the same experimental conditions. Thus, the relative comparisons of brain activation for each protocol are considered possible. Thus, we can comprehend that the mu rhythm measured in the sensorimotor cortex was the most suppressed in SCMT of CPM-MT. Therefore, the brain is more activated when SCMT is used than when CMT or ACMT is used. Moreover, we can observe that the differences in relative PSD between C3 and C4 decreased when SCMT was performed because MT is basically a bilateral rehabilitation method.

### 3.2. Muscle Fatigue Analysis: EMG

The use of CPM system may increase muscle fatigue owing to artificial movement, and the accumulation of lactic acid in muscle fatigue can cause muscle damage, local myalgia, chronic fatigue syndrome, overtraining syndrome, endocrine disorders, and organic diseases, as well as a threat to human health. Therefore, we monitored MPF and RMS, which are the parameters of muscle fatigue change, during treatment.

Muscle fatigue, which can be monitored using sEMG to measure the myoelectric activity of the biceps brachii [52], causes a decrease in MPF and an increase in RMS, originating from shifts of the PSD of the EMG signal toward relatively lower frequencies [53]. The ratio of MPF and RMS was calculated using Equation (4):

(4) $$Ratio\;of\;MPF,\;RMS=\frac{MPF,\;RMS_{after}}{MPF,\;RMS_{before}}$$

Table 3 shows the changes in the MPF and RMS ratio after each session and their absolute average. CMT, ACMT, and SCMT groups did not show a significant decrease in MPF and RMS ratio after each session (*p* > 0.05).

According to this analysis, the effect of CPM-MT system on muscle fatigue in the paralyzed arm is negligible. Thus, our CPM-MT system helps to improve patients’ motor ability by sensorimotor cortex activation with minimal negative effects of artificial movement.

To the best of our knowledge, this is the first study to demonstrate the effectiveness of sensory stimulation-based CPM-MT system in terms of a control method (asynchronous and synchronous control) and human influence (muscle fatigue). To date, there have been few studies on the modulation of EEG cortical activity during robot-assisted tasks [54,55], and no study has evaluated different CPM-MT methods and their effect on muscle fatigue.

### 3.3. Suggestions for High-Efficiency MT

Previous studies have not considered ways to find an effective method of MT. We have shown that the movement of the paralyzed arm by CPM system and synchronization of both arms are important to maximize the effect of MT by brain activation. The use of the brain activation factors found in this study will help to develop an effective rehabilitation system to restore motor function in post-stroke patients.

Table 4 shows the standard deviations for the relative PSD over the electrodes Cz, C3, and C4 according to each rehabilitation protocol. Even if the same rehabilitation protocol is applied, variation in brain activation will be observed between subjects. Thus, it is necessary to personalize studies on MT by finding which treatment conditions are more likely to affect brain activation using a closed-loop system.

It is also possible to study not only a mechanically assisted method, but also the changes that occur when the paralyzed arm is stimulated electrically, such as by functional electrical stimulation, to produce movement. Future studies may be performed to determine which stimulation parameters will have more effect on brain activation.

Using these experiments, we verified that our CPM-MT system does not cause muscle fatigue. However, depending on the experimental protocol and conditions, fatigue can occur. Thus, it is possible to develop a closed-loop system that controls the torque or operating speed of CPM-MT system by monitoring the changes in muscle fatigue in real time.

## 4. Conclusions

To improve the effectiveness of rehabilitation of post-stroke patients, we developed a sensory stimulation-based CPM-MT system using two different operating protocols called asynchronous and synchronous modes. As a pilot study to demonstrate the feasibility of the proposed system, we conducted experiments on healthy subjects and plan a later study with stroke patients.

We analyzed how the rate of PSD of the mu rhythm in the sensorimotor cortex as well as MPF and RMS related to muscle activity signals of the upper extremities are changed after three protocols, that is, CMT, ACMT, and SCMT.

The PSD of the mu rhythm is most suppressed in SCMT. Moreover, there is slight change in muscle fatigue caused by the CPM-MT system. Thus, the movements of the paralyzed arm and the timing of its movements are important determinants of activation of the sensorimotor cortex. Additionally, MT produces rehabilitation effects through action observation (AO) and motor imagery (MI) [56,57]. Action execution (AE) also plays an important role compared with AO [38,58]. However, CMT cannot perform AE of the paralyzed arm.

It is also important to provide sustained sensory input to patients who have suffered a stroke. Thus, we added the magnetic-based MT task for both arms to increase the rehabilitation effects by providing tactile stimulation. Therefore, to administer a combination treatment, we designed CPM-MT system to merge AO, AE, MI, and sensory stimulation.

In this combination rehabilitation treatment, an effective MT system is proposed that maximizes brain activation within the limited treatment time, and the effect of this system is demonstrated through EEG analysis. As a result, SCMT with sensory stimulation is the most effective method for brain activation related to motor activity; therefore, these results can be useful for the development of an effective MT system and protocol using the existing CPM machines to recover patients’ motor ability.

## Figures and Tables

**Table 1:** Ratio of relative power spectral density (PSD) analysis after each rehabilitation protocol. CMT, conventional mirror therapy; ACMT, asynchronous mode CPM−MT; SCMT, synchronous mode CPM−MT.

Rehabilitation Protocol	CMT			ACMT			SCMT		
Electrode Location	Cz	C3	C4	Cz	C3	C4	Cz	C3	C4
**Ratio of relative PSD** **after first session**	1.08	1.14	1.07	1.03	1.02	1.03	1.12	1.04	1.04
**Ratio of relative PSD** **after last session**	1.08	1.06	1.02	0.93	0.92	0.94	0.87	0.88	0.87
**Change after** **treatment (%)**	0.01	−7.00	−4.42	−9.16	−9.63	−8.03	−22.11 *	−15.31 *	−16.48 *
**Change after** **first session (%)**	9.47	15.39	8.28	0.92	0.57	0.67	5.92	−0.32	−0.87
**Change after** **last session (%)**	5.86	5.48	0.40	−8.66	−8.97	−8.02	−16.08 *	−13.51 *	−15.93 *

Ratio of first session and last session according to each rehabilitation protocol by electrode location, and changes in relative PSD ratio after each session and treatment (* *p* < 0.05).

**Table 2:** Absolute power spectral density (PSD) changes of each frequency band according to the rehabilitation protocol.

Rehabilitation Protocol	CMT			ACMT			SCMT		
Electrode Location	Cz	C3	C4	Cz	C3	C4	Cz	C3	C4
**Theta rhythm (%)**	−5.78	1.58	−2.25	3.33	3.91	5.90	−21.23	−17.87	−18.11
**Mu rhythm (%)**	−12.52	−15.34	−14.26	−9.92	−10.34	−7.65	−60.71 **	−38.08 *	−44.28 *
**Beta rhythm (%)**	−1.15	4.85	−3.81	7.40	2.79	1.25	−15.13	−1.73	−9.89
**Gamma rhythm (%)**	−1.91	2.26	−6.30	3.64	11.00	−3.40	5.50	20.19	−9.83

Absolute PSD changes of each frequency band: theta rhythm, mu rhythm, beta rhythm, and gamma rhythm (* *p* < 0.05, ** *p* = 0.05).

**Table 3:** Muscle fatigue analysis using median power frequency (MPF) and root mean square (RMS).

Rehabilitation Type	CMT		ACMT		SCMT	
	MPF	RMS	MPF	RMS	MPF	RMS
**First session (%)**	−1.66 ***	18.61 ***	2.61 ***	−11.67 ***	0.13 ***	−2.11 ***
**Last session (%)**	0.11 ***	6.80 ***	0.56 ***	−7.88 ***	−1.19 ***	6.36 ***
**Abs. avg (%)**	0.89 ***	12.71 ***	1.59 ***	9.78 ***	0.66 ***	4.24 ***

Changes in MPF and RMS ratio after the first and last treatment session and absolute average of MPF and RMS (*** *p* > 0.05).

**Table 4:** Standard deviations of relative power spectral density (PSD).

Electrode Location	Cz	C3	C4
**CMT (%)**	33.76	27.52	26.24
**ACMT (%)**	17.46	17.49	16.58
**SCMT (%)**	22.40	12.79	10.75

Standard deviations of relative PSD according to electrode location and protocol.
